# Reduced Cytosolic Calcium as an Early Decisive Cellular State in Parkinson’s Disease and Synucleinopathies

**DOI:** 10.3389/fnins.2018.00819

**Published:** 2018-11-06

**Authors:** Cristine Betzer, Poul Henning Jensen

**Affiliations:** ^1^DANDRITE – Danish Research Institute of Translational Neuroscience, Aarhus University, Aarhus, Denmark; ^2^Department of Biomedicine, Aarhus University, Aarhus, Denmark

**Keywords:** aggregation, α-synuclein, calcium, synucleinopathies, dementia

## Abstract

The more than 30-year-old Calcium hypothesis postulates that dysregulation in calcium dependent processes in the aging brain contributes to its increased vulnerability and this concept has been extended to Alzheimer’s disease and Parkinson’s disease. Central to the hypothesis is that increased levels of intracellular calcium develop and contributes to neuronal demise. We have studied the impact on cells encountering a gradual build-up of aggregated α-synuclein, which is a central process to Parkinson’s disease and other synucleinopathies. Surprisingly, we observed a yet unrecognized phase characterized by a reduced cytosolic calcium in cellular and neuronal models of Parkinson’s disease, caused by α-synuclein aggregates activating the endoplasmic calcium ATPase, SERCA. Counteracting the initial phase with low calcium rescues the subsequent degenerative phase with increased calcium and cell death – and demonstrates this early phase initiates decisive degenerative signals. In this review, we discuss our findings in relation to literature on calcium dysregulation in Parkinson’s disease and dementia.

## Introduction

**FIGURE 1:**
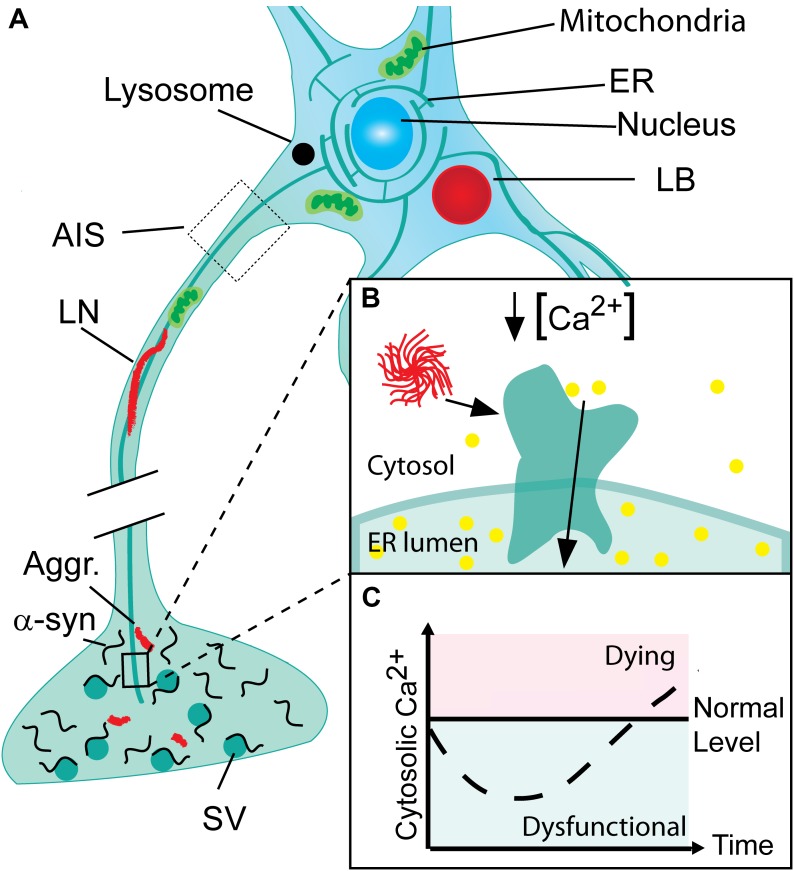
Progressive neuronal accumulation of α-synuclein aggregates in Parkinson’s disease cause gradual activation of SERCA and changes in cytosolic Ca^2+^ and Ca^2+^ dependent processes. **(A)** Nerve terminals with their high concentration of α-synuclein are the sites for the initial build-up of α-synuclein (α-syn) aggregates (Aggr.). The aggregates are transported to the cell body by active retrograde axonal transport and during this process accumulate as Lewy neurites (LN). In the cell body, aggregates may accumulate locally as Lewy bodies (LB) if not degraded by lysosomes. **(B)** The endoplasmic reticulum (ER) permeates the entire neuron into its terminals. In Parkinson’s disease (PD), α-syn aggregates bind and activate SERCA resulting in reduced cytosolic Ca^2+^ and Ca^2+^ overload in the ER. **(C)** The cytosolic Ca^2+^ level in neurons encountering a progressive build-up of intracellular α-synuclein aggregates displays a biphasic response with a reduced level in the early phase Ca^2+^ ions are translocated into the ER. This phase will be characterized by a range of dysfunctions caused by deranged Ca^2+^ dependent processes. Later the compensatory mechanisms fail and the cell progress into a degenerative state with increased Ca^2+^ that precede cell death.

In Parkinson’s disease (PD), dopaminergic neurons in the substantia nigra pars compacta have been the focus of interest for decades due to their disease-associated loss that gives rise to the striatal dopamine deficiency and motoric symptoms. An intense interest in the cytosolic Ca^2+^ levels of these cells has been based on two independent lines of evidence. First, the autonomous firing of these dopaminergic (DA) neurons along with their large terminal fields causes a continuous and large influx of Ca^2+^ that in order to maintain low normal Ca^2+^ levels has to be balanced by active transport processes at the risk of oxidative stress (Surmeier, 2007; Dryanovski et al., 2013; Surmeier et al., 2017). Second, epidemiological studies have demonstrated that treatment with L-type Ca^2+^ channel antagonists that lowers Ca^2+^ influx reduces the risk of developing PD symptoms (Ritz et al., 2010; Pasternak et al., 2012). We recently reported a novel phenotype with reduced cytosolic Ca^2+^ in neurons that occurs very early in the degenerative processes associated to PD (Figure 1). In contrast to previous hypotheses focusing on loss of cells, this observation corroborates the existence of prolonged phase with reduced Ca^2+^ in neurons that encounter progressive build-up of α-synuclein aggregates (Betzer et al., 2018). This phase may contribute to symptomatology by changing the neurons contribution to functional motor and non-motor circuitries.

## Cytosolic Calcium Ions

Calcium ions are versatile cellular second messengers and proper control of cytosolic Ca^2+^ levels in neurons are crucial for their development and function (Berridge et al., 2000, 2003). The resting cytosolic Ca^2+^ concentration in neurons is approximately 150 nM but this term is a crude measure because most Ca^2+^ signaling is mediated within subcellular microdomains (Rizzuto and Pozzan, 2006). This is particularly true for neurons that are highly polarized with separate microdomains like nerve terminals, axon initial segment and synaptic boutons. Still the experimental use of cytosolic Ca^2+^ sensors like Fura-2 and Fluo3 have given important insights into neuronal Ca^2+^ signaling despite their suboptimal spatial resolution.

The average 150 nM cytosolic Ca^2+^ contrasts the endoplasmic reticulum (ER) and the extracellular compartment where Ca^2+^ concentrations are in the 1 mM range (Berridge et al., 2003; Surmeier and Schumacker, 2013) and these steep gradients are maintained by active transport through efficient pumps and secondary active cotransporters like sarcoplasmic/endoplasmic reticulum Ca^2+^ ATPases (SERCA), plasma membrane ATPases (PMCA), and Na^+^/Ca^2+^ exchangers (NCX).

It was early recognized that dysfunctions herein triggered by a broad range of cellular stresses, e.g., decreased ATP and O_2_, per default will increase cytosolic Ca^2+^ and ultimately cause cell death (Berridge et al., 1998). However, more subtle changes in leakiness or efficiency of specific pumps could also have fundamental, but slowly progressing effects on the brains ability to compensate with aging and age-related neurodegenerative diseases as put forth in the Ca^2+^-hypothesis (Khachaturian, 1984, 1994; Berridge, 2010).

## Parkinson’s Disease and α-Synuclein

**FIGURE 2:**
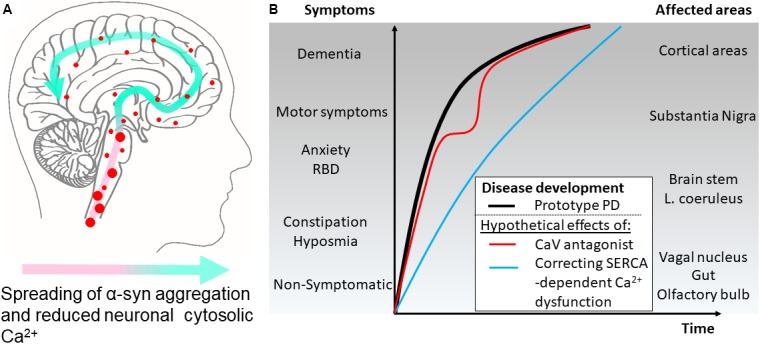
Hypothetical effects on symptomatology in Parkinson’s disease by modulating cytosolic calcium in neurons by different mechanisms. **(A)** The disease course for a Parkinson’s disease patient can be divided into different phases. According to the Braak hypothesis, the phases represent the sweeping movement of α-synuclein aggregate-dependent neuropathology through the nervous system and is initially detectable in the lower brain stem and the olfactory bulb. **(B)** The presymptomatic phase represent early aggregate build-up in the neurons of the olfactory bulb, the gut and the vagal motor nucleus of the lower brain stem and evolves into the symptomatic phase with hyposmia and constipation as frequent symptoms. Further involvement of the brain stem may add symptoms like sleep disturbances, e.g., REM sleep behavior disorders (RBD), depression, and anxiety. Next, the substantia nigra becomes involved and the patients develop motor symptoms and get diagnosed as PD patients. Finally, higher areas are involved adding cognitive problems to the disease complex. Treatment of patient with L-type Ca^2+^ channel antagonists aims at normalizing Ca^2+^ dysfunctions in the pace-making firing dopaminergic neurons of the substantia nigra pars compacta and other vulnerable neuronal populations. Epidemiological data suggest this treatment lowers the motoric symptoms but not the final progression into dementia as depicted by a red line. A treatment that targets the early Ca^2+^ dysfunctions caused by SERCA activation by α-synuclein aggregates will ideally normalize the early dysfunctions of affected neurons. This could apart from reducing the severity of non-motor symptoms also decrease the rate of disease progression through the brain (blue line).

Our insight into PD pathophysiology has developed significantly within the last 20 years since (i) mutations within the SNCA gene encoding α-synuclein was identified as causative for autosomal dominant PD in rare families (Zeng et al., 2018), (ii) aggregated α-synuclein was demonstrated as the main component in the neuronal Lewy body inclusions in PD (Spillantini et al., 1997), and (iii) the Braak hypothesis was presented (Braak et al., 2003) on PD being a progressive neurodegenerative disease that continuously involves new but selective areas of the brain thereby bringing increased complexity to the patients symptomatology. Today, it is commonly recognized that many PD patients have suffered from non-motor symptoms, e.g., depression and sleep disturbances, years before they develop motor symptoms leading to PD diagnosis (Figure 2). Hence, mechanisms applicable to larger groups of vulnerable neurons rather than the dopaminergic neurons of the substantia nigra pars compacta are attractive targets if the aim is to modify the disease course at different stages from presymptomatic to late-stage disease. The presymptomatic intervention is, as for all late-onset neurodegenerative diseases, especially attractive but requires strong biomarkers to identify at-risk patients. The spreading of pathology is hypothesized to be carried by prion-like aggregates of the normally presynaptic protein α-synuclein that upon uptake in healthy neurons cause progressive conversion of their native α-synuclein into aggregated species. The aggregated species represents a still poorly described group that initially are hypothesized to contain soluble, so-called oligomers or protofibrils, before they convert into amyloid-type filaments. The filaments are deposited in intracellular inclusions localized in axons and cell bodies as Lewy neurites and Lewy bodies that represents pathoanatomical hallmarks of PD (Figure 1). The process of α-synuclein aggregation is dose-dependent as evidenced by the autosomal dominant heritability of PD in rare families with elevated levels of normal α-synuclein caused by multiplications of the α-synuclein gene SNCA (Singleton et al., 2003; Chartier-Harlin et al., 2004; Devine et al., 2011). Moreover, GWAS studies demonstrates variations in the SNCA locus is the largest genetic risk factor for sporadic PD and this effect is hypothesized to be due to increased expression α-synuclein. α-Synuclein is thus considered a key player in the development and progression of PD although it still is unclear which intracellular α-synuclein aggregates harm the neurons and how this is accomplished. Insight into the molecular structure of *in vitro* formed α-synuclein aggregates has only recently reached the level of atomic resolution by CryoEM (Guerrero-Ferreira et al., 2018) and solid state NMR studies (Tuttle et al., 2016). The ability to validate that features of *in vitro* formed aggregates indeed exist *in vivo* has benefitted from the development of aggregate specific antibodies (Lindersson et al., 2004; Vaikath et al., 2015; Lassen et al., 2018; Peng et al., 2018) that allows the validation of specific epitopes on both types of aggregates. Biochemical and structural analyses of α-synuclein aggregates isolated from brain tissue (Gai et al., 2000; Anderson et al., 2006) or cells are sparse but specific targets for α-synuclein aggregates has been identified (Lindersson et al., 2004, 2005; Betzer et al., 2015, 2018; Mao et al., 2016).

We have previously demonstrated that decisive prodegenerative signals, like changes in gene expression and secreted signaling molecules are generated at early time points in human α-synuclein expressing immortalized rat oligodendroglial cells (OLN-93) encountering a progressive build-up of soluble α-synuclein aggregates (Kragh et al., 2009, 2013, 2014). These can be measured in the OLN-93 model at early time points, where no gross dysfunctions are apparent. Recent investigations of this early phase allowed us to demonstrate by use of the ratiometric calcium sensor, Fura-2 that it is characterized by a reduction in cytosolic Ca^2+^ in both mitotic OLN-93 and a non-mitotic human neuroblastoma cell model (SH-SY5Y) of α-synuclein aggregate stress, and in primary cultures of mouse hippocampal neurons expressing human α-synuclein from the Thy1-a-Syn Line 61 (Betzer et al., 2018). The early phase with reduced cytosolic Ca^2+^ preceded the well-known late phase with increased cytosolic Ca^2+^ and increased cell death (Figure 1). Mechanistically the decreased cytosolic Ca^2+^ was caused by binding of soluble α-synuclein aggregates to SERCA, leading to increased pumping of Ca^2+^ from cytosol into ER. The increased activity of SERCA was measured by *in vitro* assays using sarcoplasmic microsomes isolated from rabbit muscle as the source of SERCA. Here was measured an increased hydrolysis of ATP, transport of ^45^Ca^2+^, and dephosphorylation rate of the pump. Furthermore, analysis of the rate of Ca^2+^ loading into ER in the OLN-93 cells expressing the calcium sensor aequorin in ER revealed an increased uptake in the presence of α-synuclein aggregates. The Ca^2+^ dysregulation could be treated pharmacologically by the specific SERCA inhibitor cyclopiazonic acid (CPA) leading to reduced cell stress and increased viability in the OLN-93 model, SH-SY5Y model, in the hippocampal neurons, and an *in vivo* C. elegans model, UA44 [baInl1; Pdat-1::AS, Pdat-1::GFP] expressing AS and GFP in their dopaminergic neurons. The protective CPA dose did not negatively affect the viability of control cells. The abnormal complexes between α-synuclein aggregates and SERCA could be demonstrated by co-immunoprecipitation of SERCA from brain homogenates using *in vitro* formed soluble aggregates as bait and *in situ* in SH-SY5Y cells using anovel α-synuclein/SERCA proximity ligation assay. The proximity ligation assay also revealed positive signals in human brain tissue affected by dementia with Lewy bodies, but not control tissue corroboration this novel mechanism of α-synuclein aggregates engaging with SERCA is relevant to human pathology. Hence, the degenerative phase with reduced cytosolic Ca^2+^ may well be active in human brain. It should be stressed that the mechanisms, whereby α-synuclein aggregates activates SERCA is not restricted to neurons but is generic and will occur in all cells experiencing intracellular α-synuclein aggregates, since SERCA is ubiquitously expressed (Primeau et al., 2018). SERCA is expressed by three genes, ATP2A1, 2 and 3, and their isoform diversity is increased by alternative splicing, giving rise to 13 isoforms (Wuytack et al., 2002; Dally et al., 2010). The expression of SERCA isoforms exhibit both specificity with respect to developmental state and tissue, with SERCA2b and SERCA3 as the predominant isoforms in brain (Uhlen et al., 2015). It will thus apart from PD, Lewy body dementia, and Lewy body variant of Alzheimer’s disease (AD), also occur in Multiple systems atrophy, where aggregates accumulate in oligodendrocytes (Gai et al., 1998; Spillantini et al., 1998) as modeled by our OLN-93 models (Betzer et al., 2018). In PD, does astrocytes in proximity of LB containing neurons also contain α-synuclein (Braak et al., 2007) but it does not appear to be aggregated and thus able to affect their SERCA pumps (Takemiya and Yamagata, 2013).

One may ask why this phase with reduced cytosolic Ca^2+^ have not been appreciated before? Firstly, the experimental focus has been on cell models to generate inclusion or pathology that resembles Lewy bodies e.g., phosphorylation of Ser129 on cellular α-synuclein or insoluble α-synuclein species. These phenomena likely require a significant build-up of α-synuclein aggregates and may occur in the late degenerative stage with increased cytosolic Ca^2+^ and enhanced cell death. Secondly, methodologically it is not trivial to demonstrate a 20% decrease in basal cytosolic Ca^2+^ of 150 nM if it is not the aim of the experiment. The genetically encoded Ca^2+^ sensors, like GCAMP6, are designed to demonstrate increases in Ca^2+^ as readouts for neuronal activity. To obtain quantitative measurement of reductions in cytosolic Ca^2+^, we used the ratiometric Ca^2+^-sensor Fura-2 that is exited at 340 and 380 nm and has its emission quantified at 510 nm, which upon calibration allows quantitative measurements. In our study, we chose a region of interest to measure the Fura-2 signal in the cell body located outside the nucleus that may reflect neurons average cytosolic Ca^2+^ in the somatodendritic compartment.

Conclusively, when neurons and other cells, like oligodendrocytes in MSA, experience a progressive build-up of α-synuclein aggregation we propose the existence of an early phase characterized by a decreased cytosolic Ca^2+^ and a degree of Ca^2+^ overload in their ER. This phase is not characterized by degenerative death promoting processes, but slowly developing dysfunctions in critical Ca^2+^ dependent processes that compromise the cells ability to contribute to its normal circuitries and thereby brain functions. We propose a neuron-centric mechanisms where Ca^2+^ dysfunctions initiates in neurons that contain the α-synuclein aggregate but one can speculate if it also affect the tripartite synapse coupling neuron and glia (Eroglu and Barres, 2010). The so-called tripartite synapse consists of pre- and postsynaptic neuronal structures along with the astrocyte processes that connects hereto. The astrocytes contribute to the neuron-neuron signaling by releasing glial transmitters that bind to receptors on both pre- and postsynaptic neuronal structures (Eroglu and Barres, 2010). The SERCA activated dysfunctions in neurons may affect both the release of transmitters targeting astrocytes and the receptors that respond to the glial transmitters. Insight into these processes may allow direct targeting the abnormally activated SERCA pump or its downstream signaling pathways. This reorientation of focus from what kills the cells to what make them dysfunctional, may allow us to relieve symptoms caused by dysfunction of neurons and/or glia. Nerve cells often function as parts of closely regulated circuits that by support from glia cells contribute to brain functions and dysfunctions herein cause symptoms. A focus on alleviating cellular dysfunctions may open for more points of therapeutic intervention.

## Evidence of Neuronal Calcium Disturbances in PD

The Ca^2+^ hypothesis that originally was associated to aging (Khachaturian, 1984) and later AD (Berridge, 2010) has also rationally been adopted in the PD field. First, it was demonstrated that dopaminergic neurons in the substantia nigra pars compacta display autonomous pace-making firing and this combined with their extremely large terminal fields, results in a large influx of Ca^2+^ via L-type voltage-gated Ca^2+^ channels (CaV) (Chan et al., 2007). Maintaining low normal Ca^2+^ levels by active transport processes requires large ATP production by oxidative phosphorylation and carries a risk of oxidative stress (Surmeier, 2007; Dryanovski et al., 2013; Surmeier et al., 2017). The CaV1.3 is largely responsible for the Ca^2+^ influx in pace-making neurons in substantia nigra and has been proposed to be involved in Ca^2+^ dysregulation since it does not fully close during autonomous firing (Wilson and Callaway, 2000; Bean, 2007; Puopolo et al., 2007; Surmeier and Schumacker, 2013).

Second, L-type Ca^2+^ channel blockers of the dihydropyridine type (DiCCBs) used to treat hypertension reduce the risk of developing PD and its associated mortality, but did not reduce the risk of dementia in PD patients (Becker et al., 2008; Ritz et al., 2010; Pasternak et al., 2012). The absent effect on cognition combined with a lack of association between length of previous use of Ca^2+^ channel blockers and its preventive effect, which made the authors suggest “any clinical effects of DiCCBs may be associated with symptomatic relief (preventing the development of clinical symptoms of early disease) rather than having a long-term impact on neurodegeneration” (Pasternak et al., 2012). However, changes to CaV channel expression has been reported in PD brains suggesting where a dysregulation was demonstrated by immunohistochemistry in early and late stage PD brains (Hurley et al., 2013). The study demonstrated abnormalities in CaV subtype expression with a general increase preceding PD pathology along with a change in the ratio of CaV1.2 vs. CaV1.3 to favor a greater utilization of CaV1.3 channels (Hurley et al., 2013). The mechanism governing these changes, which may allow increased Ca^2+^ influx are unknown, but could in principle be a response to decreased cytosolic Ca^2+^ in neurons. Clinical trials testing the Ca^2+^-hypothesis in PD are ongoing where the efficacy of CaV1.3 channel antagonist, Israpidine, is being evaluated^1^. However, even if the trials succeed in demonstrating symptomatic relief, they may not modify the progressive and widespread pathology that develops during the course of a PD patients life and which may be caused by prion-like properties of spreading α-synuclein aggregates (Braak et al., 2003).

## Is Increased Cytosolic Calcium Always Bad or Can It Even Be Neuroprotective?

We demonstrated that development of α-synuclein aggregates, by activating a Ca^2+^ pump, causes a decrease in cytosolic Ca^2+^. This demonstrates that not only increased Ca^2+^, as posited by the Ca^2+^-hypothesis, but also low Ca^2+^ can have adverse effects (Betzer et al., 2018). Considering the Ca^2+^-hypothesis, one may ask if increased cytosolic Ca^2+^ always is cytotoxic? Early studies focusing on developmental aspects of primary cultures of peripheral neurons demonstrated increasing intracellular Ca^2+^ levels by chronic depolarization was cytoprotective (Scott and Fisher, 1970; Franklin et al., 1995). This could also be demonstrated *in vivo* where activation of spiral ganglion neurons promoted their survival in the inner ear, which was prevented by blocking Ca^2+^ influx through L-type Ca^2+^ channels by Verapamil (Miller et al., 2003). Treatment with Verapamil was among the CaV channel antagonists that reduced the risk of developing PD symptomatology (Pasternak et al., 2012). A different neuroprotective mechanism has been demonstrated relying on synaptic stimulation of NMDA receptors (NMDAR) (Zhang et al., 2007). The NMDAR stimulation on dendritic spines, but not on extrasynaptic sites, triggers a signaling cascade that causes Ca^2+^ transients in the nucleus. The increase in nuclear Ca^2+^ stimulates transcription of specific genes, among some designated neuronal shield genes that contribute to enhanced neuronal survival and differentiation (Mao et al., 1999; Hardingham et al., 2002; Lee et al., 2005; Papadia et al., 2005; Bok et al., 2007; Zhang et al., 2007; Hardingham, 2009; Bading, 2013). In primary cultures of hippocampal neurons from α-synuclein transgenic mice, we measured the decreased cytosolic Ca^2+^ in the somatodendritic compartment just outside the nucleus (Betzer et al., 2018). Considering the fenestrated nature of the nuclear membrane this will likely result in a decreased nuclear Ca^2+^ that may blunt the protective Ca^2+^ transient elicited by synaptic NMDAR signaling.

## Calcium Overload of the Endoplasmic Reticulum

When α-synuclein aggregates activate SERCA, the transport of Ca^2+^ into the lumen of the ER is increased (Betzer et al., 2018). However, it is unclear how much this increases the Ca^2+^ load and the consequences hereof. Still it is becoming increasingly evident that the level of Ca^2+^ load in the ER plays fundamental roles for a range of important cellular processes. These processes comprise the unfolded protein response, the critical Ca^2+^ filling of mitochondria needed for oxidative phosphorylation, store-operate Ca^2+^ entry (SOCE) of Ca^2+^ across the plasma membrane into the ER (Carreras-Sureda et al., 2018) and the recently described regulation of presynaptic neurotransmitter release probability by axonal and presynaptic ER Ca^2+^ filling (de Juan-Sanz et al., 2017), and specialized overload channels like TMCO1 has been identified that counteracts ER overload (Wang et al., 2016). The significance in these pathways in neurons encountering a gradual build-up of α-synuclein aggregates needs further investigations.

## Calcium Dysregulation and Mitochondria

ER represents the major Ca^2+^ storage organelle but other organelles also play important roles in cellular Ca^2+^ homeostasis including mitochondria, Golgi apparatus, secretory vesicles, lysosomes, and peroxisomes (Rodriguez et al., 1997; Pinton et al., 1998; Mitchell et al., 2001; Saris and Carafoli, 2005; Rizzuto and Pozzan, 2006; Drago et al., 2008; Lasorsa et al., 2008). This suggests that α-synuclein aggregate-dependent stimulation of SERCA may affects the functionality of these organelles by the increased ER Ca^2+^-load and reduced cytosolic Ca^2+^ level.

Mitochondria has for long been considered a key player in PD pathogenesis as evidenced by their decreased complex 1 activity in PD patients (Schapira et al., 1989), the induction of a PD phenotype by the mitochondrial toxin MPTP (Langston et al., 1983), and the identification of mitochondria related PARK2 and PARK4 genes causing autosomal recessive PD (Kitada et al., 1998; Valente et al., 2004). Ca^2+^ influx into mitochondria is carried by diffusion through the voltage-dependent anion channel 1 (VDAC) in the outer mitochondrial membrane and through the mitochondrial Ca^2+^ uniporter (MCU) in the inner mitochondrial membrane (Bathori et al., 2006; Tan and Colombini, 2007). The efficiency of this process rely on the positioning of mitochondria in the proximity of the Ca^2+^-rich ER by means of mitochondrion-associated membrane (MAM) domains (Rizzuto et al., 2009). An estimate of up to 20% of the mitochondrial surface is in close proximity with the ER (Rizzuto et al., 1998) with a distance of 9–30 nm (Goetz and Nabi, 2006; Csordás et al., 2006). This correspond to the distance of protein complexes tethering the organellar membranes (Perkins et al., 1997). A tethering complex is formed between ER Ca^2+^-channel, inositol 1, 4, 5-trisphosphate receptor (IP3R) and VDAC1 in the mitochondria by the action of the chaperone GRP75 and this facilitates the flux of Ca^2+^ from ER into mitochondria. Mitochondria are rich in the lipid cardiolipin that facilitates their α-synuclein binding (Nakamura et al., 2011), and MAM is rich in negatively charged phospholipids (Hayashi and Fujimoto, 2010) that also facilitates binding of α-synuclein (Davidson et al., 1998). The membrane binding ability of α-synuclein and localization to MAM is abolished in α-synuclein carrying the familial PD mutation A30P (Jensen et al., 1998; Guardia-Laguarta et al., 2014) and this is associated with decreased MAM activity and mitochondrial-ER interface (Guardia-Laguarta et al., 2014). This suggests a physiological function of native α-synuclein in facilitating optimal ER-mitochondrial interactions. Aberrant forms of α-synuclein have been demonstrated to affects mitochondrial function by disrupting the Ca^2+^ traffic at ER-mitochondrial interphases (Cali et al., 2012; Guardia-Laguarta et al., 2014; Raffaello et al., 2016) and to increase ER and mitochondrial stress (Smith et al., 2005; Colla et al., 2012; Melachroinou et al., 2013). This may be caused by direct interactions between aberrant α-synuclein forms and VDAC1 (McFarland et al., 2008; Martin et al., 2014; Betzer et al., 2015). However, the significance of these findings and if they only are active in certain species, cellular states and domains has been stressed by recent studies. Mouse neurons lacking α-synuclein alone and all three synuclein forms did not demonstrate significant changes to mitochondrial bioenergetics (Pathak et al., 2017) whereas human iPSC derived neurons expressing mutant α-synuclein displayed mitochondrial dysfunctions (Ryan et al., 2018). Hence, understanding the significance of α-synuclein species interactions with mitochondria and MAM and their relation to the Ca^2+^ homeostasis of the involved organelles needs further investigations.

## α-Synuclein and Axonal Dysfunctions in PD.

α-synuclein is predominantly a presynaptic protein and its intraneuronal aggregation is likely initiated at this site (Kramer and Schulz-Schaeffer, 2007), which make the axonal transport regulating its anterograde and retrograde transport of key importance. How α-synuclein aggregate dependent SERCA activation affects these processes are currently unknown, but the fundamental role of the Ca^2+^ content in the axonal ER makes α-synuclein aggregation in this compartment especially critical (de Juan-Sanz et al., 2017). Moreover, α-synuclein holds potential of affecting other axonal functions in PD. Axonal pathology is recognized in PD as swellings crowded with organelles shown by electron microscopic studies suggestive of compromised axonal flux (Vital et al., 2014). These finding are corroborated by kinetic analyses of axonally transported biomarkers in cerebrospinal fluids from PD patients (Fanara et al., 2012). The axonal accumulation of aggregated α-synuclein in small, larger granules, and extended inclusions is the defining characteristic of Lewy neurites (Braak et al., 1999) that also can contain other axonally transported cargo like β-synuclein (Galvin et al., 1999) and such abnormalities have also been modeled in α-synuclein transgenic mice (Games et al., 2013). The functional consequences of axonal α-synuclein aggregates are not clear, but transgenic overexpression in neurons demonstrate dysfunctions in vesicle transport, e.g., of Rab7 containing endosomes (Volpicelli-Daley et al., 2014; Koch et al., 2015). α-synuclein containing axonal structures associate to both anterograde kinesin-labeled and retrograde dynein-labeled structures in neurons (Utton et al., 2005) and early reductions in kinesin levels preceded reductions reduction of tyrosine hydroxylase in the dopamine neurons (Chu et al., 2012). Hypothesizing α-synuclein aggregation predominantly initiates in the nerve terminals makes retrograde α-synuclein transport processes of pivotal importance for the clearance (Jensen et al., 1999; Bieri et al., 2018). The retrograde transport is generally considered as an active transport of endosomes and autophagosomes pulled along the microtubules by the dynein motor complex and the process has achieved increased interest because of its involvement in transport of signaling complexes from nerve terminals (Zahavi et al., 2017). α-Synuclein aggregates are able to directly target dynein complex components (Betzer et al., 2015) that may compromise retrograde transport and represent a basis for the accumulation of aggregated α-synuclein in Lewy neurites. However, α-synuclein may also affect other axonal mechanisms by e.g., directly affecting microtubule structures (Alim et al., 2004; Toba et al., 2017), and ER, and mitochondria, which both are organelles present in axons and nerve terminals (Gonzalez and Couve, 2014; Smith and Gallo, 2018). In PD, α-synuclein aggregates accumulate directly on ER (Colla et al., 2012) where they can activate SERCA (Betzer et al., 2018), but they can also bind the ER protein, VAPB (Betzer et al., 2015) thereby disrupting the ER-mitochondria tethering and affecting ATP production (Paillusson et al., 2017). Mechanistic studies that will allow a prioritization of these many interactions and mechanisms are in high demand to allow identification of potential disease modifying targets.

## Alzheimer Disease and Calcium Overload of the Endoplasmic Reticulum

The Ca^2+^-hypothesis has been widely embraced in the AD field where the moderate age-related Ca^2+^ remodeling is hypothesized to be pushed into severe Ca^2+^ signal remodeling by components of the amyloidogenic pathway central to AD (Berridge, 2010). Presenilins has turned out as central players in this model. Presenilins 1 and 2 are ER transmembrane proteins that regulate production of amyloidogenic Aβ peptides and causes familial AD in families when mutated (Tolia and De Strooper, 2009). Presenilins are, like aggregated α-synuclein, activators of SERCA pumps (Green et al., 2008). This activity, which tend to increase ER Ca^2+^ levels, is balanced by the presenilins inherent function as low conductance Ca^2+^-leak channels that allows the Ca^2+^ ions to flow back into the cytosol (Tu et al., 2006). AD causing mutations in presenilin 1 blocks this Ca^2+^-leak activity thereby causing ER Ca^2+^-overload with detrimental effects on ER-dependent signaling affecting store operated Ca^2+^ entry (SOCE), expression of ryanodine receptors (RyR), and inositol trisphosphate receptors (IP3R) (Blalock et al., 2003; Emilsson et al., 2006; Bezprozvanny, 2009; Popugaeva and Bezprozvanny, 2014). This bears resemblance to the situation that we have described for α-synuclein aggregates. Their activation of SERCA will also tend to increase ER Ca^2+^-load and may therefore share some of these neurobiological effects. It will be motivated in α-synuclein-aggregate dependent models to directly investigate if RyR. IP3R and SOCE functions are affected. SOCE has recently been demomstrated to play a central role in regulating the release probability in nerve terminals that are rich in α-synuclein (de Juan-Sanz et al., 2017).

## Hypothesis for Neuronal Dysfunctions Generated by α-Synuclein Aggregates Stimulation of Serca

Aggregation of α-synuclein in neurons is a progressive process sculpted by the local concentration of α-synuclein, presence of prion-like α-synuclein seeds, and proteostatic balances. When early soluble aggregated α-synuclein species are formed, they will bind to and activate the SERCA pump in the ER. Blocking the α-synuclein aggregate dependent activation by SERCA antagonists protected the affected neurons, thus demonstrating the existence of pivotal downstream signaling pathways. The ER is a dynamic organelle that permeates neurons from nerve terminals through axons, cell bodies into dendritic spines (Toresson and Grant, 2005) and this opens for several independent dysfunctions separated in space and time that contribute to the neurodegenerative process (Figure 1). Further insight into the contribution of SERCA-activated Ca^2+^ dysfunctions will open for novel mechanisms to counter the dysfunction associated to PD and other synucleinopathies.

## Author Contributions

CB and PJ designed and wrote the manuscript.

## Conflict of Interest Statement

The authors declare that the research was conducted in the absence of any commercial or financial relationships that could be construed as a potential conflict of interest.

## Footnotes

1 https://www.clinicaltrials.gov/
